# Morphological Evolution and Damping Properties of Dynamically Vulcanized Butyl Rubber/Polypropylene Thermoplastic Elastomers

**DOI:** 10.3390/polym14132740

**Published:** 2022-07-04

**Authors:** Qi Tang, Shiteng Hu, Lijing Han, Chengzhong Zong, Jujie Sun

**Affiliations:** 1 School of Polymer Science and Engineering, Qingdao University of Science and Technology, Qingdao 266042, China; hushiteng@qust.edu.cn; 2 Key Laboratory of Rubber-Plastics, Ministry of Education/Shandong Provincial Key Laboratory of Rubber and Plastics, Qingdao University of Science and Technology, Qingdao 266042, China; hanlijing@qust.edu.cn (L.H.); zongchengzhong@qust.edu.cn (C.Z.)

**Keywords:** thermoplastic vulcanizate, dynamic vulcanization, butyl rubber, polypropylene

## Abstract

We successfully prepared butyl rubber (IIR)/polypropylene (PP) thermoplastic vulcanizate (IIR/PP-TPV) for shock-absorption devices by dynamic vulcanization (DV) using octyl-phenolic resin as a vulcanizing agent and studied the morphological evolution and properties during DV. We found that the damping temperature region of the IIR/PP-TPV broadened with the disappearance of the glass transition temperature (T_g_) in the PP phase, which is ascribed to the improvement of compatibility between the IIR and PP with increasing DV time. As DV progresses, the size of the dispersed IIR particles and the PP crystalline phase decreases, leading to the formation of a sea–island morphology. After four cycles of recycling, the retention rates of tensile strength and elongation at break of the IIR/PP-TPV reached 88% and 86%, respectively. The size of the IIR cross-linking particles in the IIR/PP-TPV becomes larger after melt recombination, and the continuous PP phase provides excellent recyclability. Significantly, the prepared IIR/PP-TPV exhibits excellent recyclability, high elasticity, and good damping property.

## 1. Introduction

Recently, thermoplastic vulcanizate (TPV) materials have attracted much attention because of environmental issues [1]. TPVs are a special class of thermoplastic elastomers (TPEs) prepared by dynamic vulcanization (DV), a special reactive polymer-blending technology [2]. DV is a complex method of melt-blending rubber and plastic while selecting suitable cross-linking agents to drive the rubber to form a three-dimensional network structure at a high temperature. Not all rubbers and plastics can be made into TPV. The preparation of TPV needs to meet the following three conditions [3,4]: (1) The crystallinity of plastic should be >15%. (2) The critical entangled molecular weight of rubber should be as low as possible. (3) Rubber and plastic can be compatible or partially compatible, or their surface energies can be matched. Because TPV has the good elasticity of traditional cross-linked rubber at room temperature and good melt processability and recyclability of plastic at high temperatures, it has become the fastest-growing elastomer to replace non-recyclable petroleum-based thermoset rubber, and it has been widely used in te automotive, construction, electronic, and other industries [5,6,7].

Traditional rubber is a kind of non-recyclable elastomer with a three-dimensional cross-linking network structure prepared by vulcanization under static conditions (a particular time, temperature, and pressure) [8]. Compared with static vulcanized rubber, TPV is prepared by the DV method. DV is a key factor in preparing high performance TPV, which requires higher technical requirements and complicated processes [9]. The high content of rubber is broken into micron cross-linking particles by strong shear force and high temperature, which are dispersed in the low content of the continuous plastic phase to form TPV with sea–island morphology. The rubber in TPV is the chemically cross-linked soft segment, and the plastic is the physically interacting hard segment [10]. Chemically cross-linked (vulcanized) rubber embedded in the plastic matrix primarily provides elasticity, toughness, flexibility, and unique modulus [11]. The continuous plastic in TPV provides the matrix with good processability, injection, extrusion, and repeat processability. The most important properties of TPV are elasticity, melt processability, and recyclability, all of which depend on the microstructure of TPV [12]. Specifically, the content and degree of the cross-linking of rubber, the content and crystallinity of plastic, the particle size of rubber cross-linking, and the thickness of the continuous phase play key roles in the properties of TPV. The phase inversion of rubber phase from continuous phase (in premix) to dispersed phase (in TPV) occurs during DV. Therefore, the evolution of the morphology and properties of TPV during DV attracted much attention.

Butyl rubber (IIR)/PP-TPV has received much attention in recent years for its applications in shock-absorption and sound-insulation devices [13,14]. Compared with traditional materials prepared by thermoset IIR, the shock-absorption materials prepared by IIR/PP-TPV have many advantages. For example, cushioning material prepared by IIR/PP-TPV is environmentally friendly and comfortable, because the vulcanizing agent is usually phenolic resin, and no toxic gas is released during the dynamic vulcanization process [15]. Meanwhile, IIR/PP-TPV can improve production efficiency and save energy because TPV is easy to process and shape. In addition, IIR/PP-TPV can save raw materials because it is easy to recycle. In this study, we selected octyl-phenolic resins as the vulcanizing agent and prepared IIR/PP-TPV using the DV method. The cross-linking degree and the variations of the size of IIR during DV, the mechanism of phase inversion, crystal morphology, and structural evolution of the PP, and the damping properties of the IIR/PP-TPV were evaluated. Our main purpose was to understand the microstructure, damping properties, and microstructure properties of IIR/PP-TPV and to provide guidance for the preparation of high performance IIR/PP-TPV in production.

## 2. Materials and Methods

### 2.1. Materials

The experiments were carried out with IIR Butyl 301 (Lanxess Chemical, Cologne, Germany), in which the unsaturation was 1.85 ± 0.2 mol%. Butyl 301 has a density of 0.92 g/cm^3^ and a Mooney viscosity ML_(1+8)_ of 51 at 125 °C. PP4220 has a specific gravity of 0.89 g/cm^3^, melting temperature of 163 °C, and melt flow rate of 0.36 g/10 min (210 °C/2.16 kg). It was supplied by Sinopec Yanshan Petrochemical Company, Beijing, China. Pentae-rythritol tetrakis 3-(3,5-di-tert-butyl-hydroxyphenyl) propionate (Irganox 1010, Shandong Lanhai Industry Co., Ltd., Heze, China) was used as an antioxidant, and it was manufactured by Shanghai Shanpu Chemical Co., Ltd., Shanghai, China. P-(1,1,3,3-tetramethylbutyl)-phenol (RT4201) was used as a curing agent, and it was manufactured by the Shengquan industry company, China. Stannous chloride dihydrate (SnCl_2_•2H_2_O) (purity > 98%) was used as a cure accelerator and was obtained from Tianjin Beilian Fine Chemical Development Co., Ltd., Tianjin, China.

### 2.2. Preparation of IIR/PP-TPV

IIR/PP-TPV was prepared in a torque rheometer (Harbin Hapu Electric Technology Co., Ltd., RM-200C, Harbin, China) equipped with counter-rotating rotors. PP was dried in a vacuum oven at 100 °C for about 12 h before being used. The mass ratio of IIR/PP was 60/40. PP and Irganox 1010 were first melt-blended in a torque rheometer at 180 °C and 60 rpm/min for 2 min, then IIR was added, and this was mixed for another 3 min to obtain rubber–plastic blends. Subsequently, the blends were transferred to another two-roll mill (Baolun Precision Testing Instrument Co., Ltd., BL-6175-BL, Qingdao, China) at ambient temperature, and RT4201 (10.0 wt% based on IIR rubber) and SnCl_2_•2H_2_O (2.0 wt% based on RT4201) were added to obtain IIR/PP premix. The IIR/PP premix was dynamically vulcanized in the torque rheometer at 190 °C at a rotor speed of 80 rpm to obtain IIR/PP-TPV. Seven samples with various degrees of vulcanization were selected at DV times according to the torque–time curve.

### 2.3. Characterization

The cross-linking degree of the samples were tested by using the NMR Cross-link Density Meter (IIC, XLDS-15 HT, Blieskastel, Germany) at 90 °C. The samples were rectangular shaped, with dimensions of 8 mm × 5 mm × 2 mm. Frequency was set at 15 MHz, and magnetic induction intensity was 3.5 A/m. The times of applying 90° and 180° pulses were 2 ms and 4 ms, respectively. The phase structure of IIR/PP-TPV and the size of IIR cross-linking particles coated with gold were observed using a scanning electron microscope (JEOL, JSM-7500F, Akishima City, Japan) at an accelerating voltage of 20 KV. The samples were fractured in liquid nitrogen prior to microscopy. Morphological observations on crystallites of the PP phase were conducted with a polarized optical microscope (Nanjing XPT-7, Nanjing, China) at room temperature. The samples were first melted at 180 °C between two glass slides and held for 10 min to achieve thermal equilibrium. Then, they were rapidly cooled to 120 °C and isothermally crystallized for 20 min. The crystallinity of IIR/PP-TPV was measured on an X-ray diffraction analyzer (Bruker, d8 Advance, Munich, Germany) at room temperatures of 40 kV and 40 mA. The measurement speed was 5/min, and the angle 2θ was 0–50°. The dynamic mechanical properties of the samples were tested by using a dynamic mechanical analyzer (TA Instrument, Q800, Newcastle, DE, USA) under tensile mode. Frequency was set at 10 Hz and heating rate was 3 °C/min. The scanning temperature was ranged from −70 °C to −80 °C. The samples were rectangular shaped with dimensions of 15 mm × 4 mm × 2 mm. Mechanical properties of the samples were performed on a universal testing machine (Zwick Z005, Ulm, Germany) according to the ASTM D 412 standard. All tests were conducted at ambient temperature (23 °C) at a fixed crosshead speed of 500 mm/min and five specimens were tested for each composition. The dispersion morphology of the samples was observed under a transmission electron microscope (JEOL, JEM-1200EX, Tokyo, Japan) at an accelerating voltage of 200 KV. Ultrathin sections of sheets were cut at about −100 °C using an ultramicrotome (LEICA, EM-FC7, Wetzlar, Germany) equipped with a diamond knife for TEM investigations.

## 3. Results

### 3.1. Evolution of Cross-Linking Degree of IIR during DV

**Figure 1:**
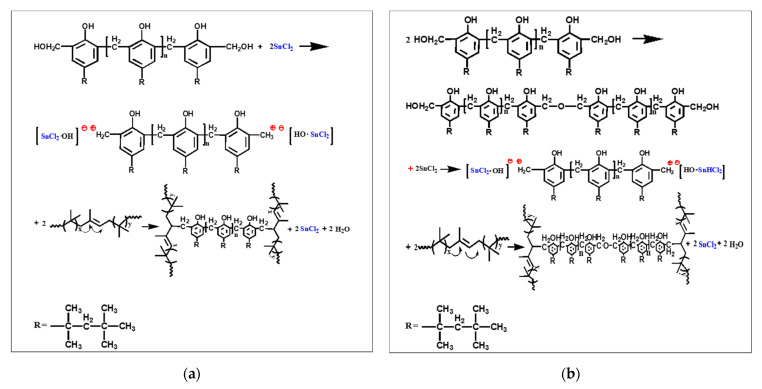
Mechanism of IIR vulcanized by octyl-phenolic resin: (**a**) mechanism of formation of C–C. (**b**) mechanism of formation of C–O–C.

In the DV process, the cross-linking degree is related to tensile strength and plays an important role in the morphological evolution of TPV [16]. Figure 1 shows the cross-linking mechanism of IIR with octyl-phenolic resin (RT4201) and stannous chloride dihydrate as vulcanizing agents [17]. RT4201 reacts with the double bond of isoprene in IIR to form stable –C–C– and –C–O–C– cross-linked bonds without reversion. Compared with peroxides, the octyl-phenolic resin does not generate free radicals and has no degradation effect on PP, which ensures the mechanical properties of IIR/PP-TPV [18].

**Figure 2:**
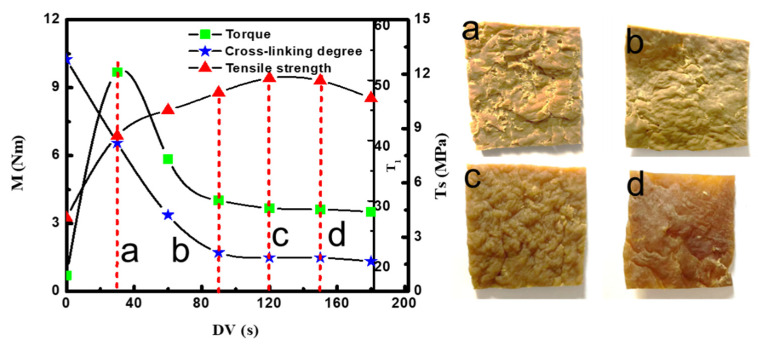
The evolution of torque (M), spin–lattice relaxation (T_1_), and tensile strength (T_s_) of IIR/PP-TPV with different DV times in torque rheometer. Samples with different DV times: (**a**) 30 s, (**b**) 90 s, (**c**) 120 s, and (**d**) 150 s.

Figure 2 shows the evolution of torque (M), spin–lattice relaxation (T_1_), and tensile strength (T_s_) of IIR/PP-TPV in a torque rheometer at different DV times. The degree of vulcanization can be probed via the spin–lattice relaxation time (T_1_) measurement and by the dipolar correlation effect [19]. The smaller the value of T_1_, the greater the cross-linking degree of the polymer. Figure 2 shows that the cross-linking degree increases rapidly at DV (<40 s), indicating that the vulcanization of IIR mainly occurs at the early stage of the DV process. T_s_ increases first; then, it tends to be flat. At the beginning of DV, RT4201 and SnCl_2_·2H_2_O rapidly react with IIR by high temperature and strong shearing force. The cross-linking degree of IIR gradually increased; therefore, the T_1_ value gradually decreased. When the DV time reaches 90 s, the cross-linking reaction is completed and the T_1_ value tends to be constant. As can be seen from Figure 2, the color of the sample gradually becomes darker as DV progresses, which also indicates that the cross-linking degree of IIR gradually increases. When DV time reaches 120 s, the T_s_ of IIR/PP-TPV reaches its maximum value of 11.98 MPa. The cross-linked network in IIR/PP-TPV is mainly composed of –C–C– and –C–O–C–, which has high bond energy and good aging resistance [20]. Therefore, at the end of DV, T_s_ does not decrease after reaching the maximum value. The torque (M) first increases and then decreases, and finally, it becomes stable. Because of the rapid cross-linking of IIR in the early stage of DV, the modulus and torque increase rapidly. When the cross-linking degree of the IIR reaches 40 s, the rubber and plastic begin to reverse. The dispersed PP transforms into the continuous phase, and the modulus and M decrease. M remains constant until the cross-linking particles of the IIR are destroyed to a certain extent. The results show that the change of M is not completely dependent on the cross-linking degree but is also related to the morphological evolution of the rubber/plastic phase during DV.

### 3.2. Morphological Evolution of Rubber/Plastic Phase of IIR/PP-TPV during DV

**Figure 3:**
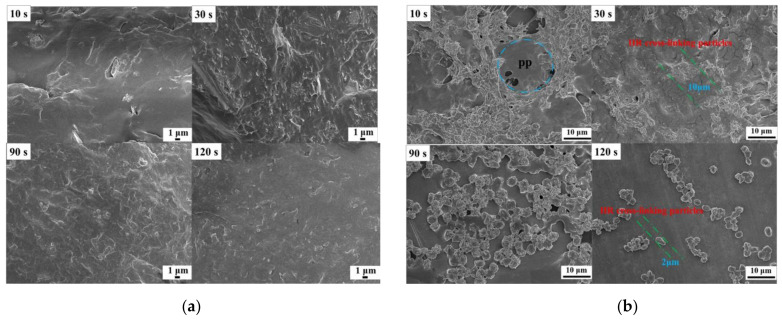
The SEM micrographs: (**a**) IIR/PP-TPV with different DV times; (**b**) cross-linking particles of IIR after samples dissolved in hot xylene (120 °C × 6 h).

During the DV process, the rubber phase is dispersed in the plastic matrix by means of high temperature and strong shearing force, and the size and distribution of cross-linking particles have an important influence on the performance of IIR/PP-TPV [21]. Figure 3a shows the fracture morphologies of the samples at 10 s, 30 s, 90 s, and 120 s during DV processes. Figure 3b shows the micromorphology of the IIR cross-linking particles extracted with xylene under solution heating. The light parts are the cross-linking particles of the IIR, and the dark parts are the tin foil on the sample. Figure 3b shows that, at the initial stage of DV (10 s), the PP etched by xylene presents various irregular spherical shapes with a diameter of about 15 μm. Due to the low cross-linking degree, the high content of the IIR surrounds the PP phase, which can be melt-reformed after crushing. When DV reaches 30 s, sea–island morphology transforms into the continuous phase, and the IIR phase is broken into irregular cross-linking particles with 10 μm. At this time, the continuous phase of the IIR/PP-TPV began to reverse, and the fragmentation and melt recombination of the IIR cross-linking particles occurred simultaneously [22]. When the DV reaches 90 s, the microscopic phase of the blend forms sea–island morphology. The IIR cross-linking particles are the dispersed phase, and the PP phase is the continuous phase. A higher degree of cross-linking, higher shear rate, and moderate cross-linking rate of the IIR phase facilitate the rapid formation of the IIR cross-linking particles and accelerate the occurrence of faster phase inversion. When the phase reverse was completed, the diameter of the IIR cross-linking particles decreased to about 2 μm. This occurs primarily because, as the cross-linking degree increases, the viscosity of the IIR cross-linking particles increases (cannot be melt-compounded), and they disperse evenly in the PP phase. When the DV time reaches 120 s, the diameter of the IIR cross-linking particles does not change much, but the dispersion is more uniform.

### 3.3. Evolution of Crystal Morphology and Structural Evolution of PP during DV

**Figure 4:**
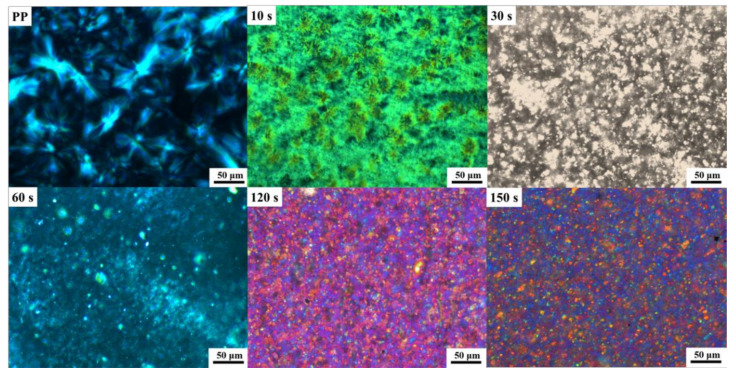
POM diagrams of IIR/PP-TPV samples with different DV times.

To study the evolution of crystal morphology and the structural evolution of PP during DV, the phase morphology of samples A to F was investigated by using POM, and the results are shown in Figure 4. Figure 4 shows that the PP in the IIR/PP-TPV with different DV times exhibits an obvious spherulite structure. As DV time increases, the crystal size of the PP phase decreases, and the distribution becomes more uniform. This occurs primarily because IIR is broken into IIR cross-linking particles with a size of 2 μm during the DV process. The IIR cross-linking particles act as a heterogeneous nucleating agent and are uniformly dispersed in the PP phase matrix to refine the PP crystal size.

**Figure 5:**
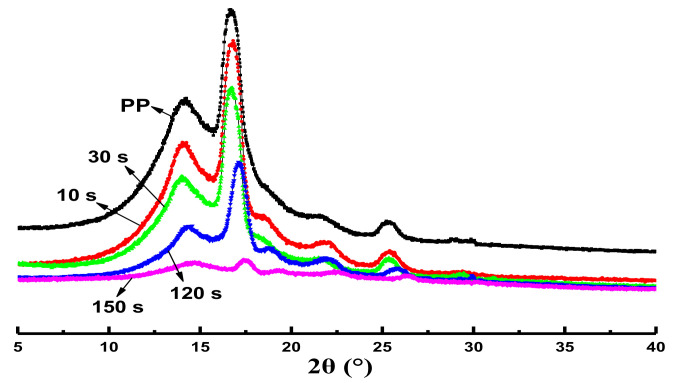
XRD pattern of IIR/PP-TPV samples with different DV times.

To further investigate the crystal structure of the PP phase, Figure 5 shows the XRD pattern of pure PP and IIR/PP-TPV at different DV times. Figure 5 shows that with the increase in DV time, the diffraction peak intensity (2θ) of the PP phase in the IIR/PP-TPV gradually decreases, but the position of the main diffraction peak remains unchanged. The appearance of 2θ occurs at 14.1°, 16.7°, 18.6°, 21.7°, and 25.3°, respectively, belonging to the α monoclinic crystal [23]. The DV of IIR/PP-TPV is the polycondensation reaction of octyl-phenolic resin with the double bond of isoprene in IIR, without any chemical reaction with the PP phase. Therefore, the macromolecular chain of the IIR is not inserted into the crystallization region of the PP phase and does not destroy the crystal structure of the PP phase. The morphological evolution of the IIR and PP phases during the DV process has an important influence on the crystallization process of the PP phase. With the increase in DV time, the size of IIR cross-linking particles gradually decreased and dispersibility increased, which hindered the crystallization of PP [24]. The IIR cross-linking particles formed reduced the crystallinity of PP, thereby reducing its intensity.

### 3.4. Evolution of Damping Properties of IIR/PP-TPV during DV

**Figure 6:**
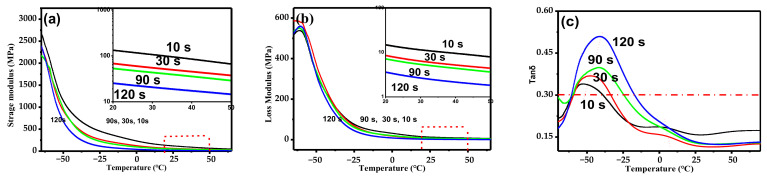
DMA thermogram of IIR/PP-TPV with different DV times: (**a**) E′, (**b**) E″, and (**c**) Tan δ.

There are many methyl groups on the macromolecular chain of IIR, leading it to possess many special properties. In the glass transition region (T_g_), the storage modulus (E′) of IIR is significantly reduced compared with the loss modulus (E″), showing obvious damping performance [25]. The DMA thermogram of IIR/PP-TPV under different DV times is shown in Figure 6. It can be seen from Figure 6a that the E′ of the IIR/PP-TPV decreases rapidly around −45 °C because the macromolecular segment of the IIR is frozen at T_g_. The E′ of IIR/PP-TPV (DV time between 10 s and 30 s) decreases rapidly near 6 °C, which is the T_g_ of the amorphous region of the PP phase. When the temperature is higher than 25 °C, the E′ of the IIR/PP-TPV changes little with the increase in temperature, mainly because the chain segment in the IIR can move freely in the rubbery plateau. In order to identify the behavior of materials above 25 °C, we plot E′ from 25 °C to 50 °C by log scale in Figure 6a. It can be seen from Figure 6a that E′ gradually decreases with the increase in DV time after 25 °C, which is mainly caused by the increase in the IIR cross-linking degree. The change trend of loss modulus E″ is the same as that of E′.

The ratio of E″ to E′ is the loss factor (Tan δ). The Tan δ of IIR/PP-TPV under different DV times is shown in Figure 6c. The peak of Tan δ represents T_g_, which can also characterize the compatibility of different polymers. The T_g_ of the IIR/PP-TPV at different DV times obtained from Figure 6c is shown in Table 1. It shows that the samples have two tan δ peaks, when the DV time reaches 10 s and 30 s. The first strong tan δ peak (at about −45 °C) is the T_g_ of IIR, and the other weak tan δ peak (at about 5 °C) is the T_g_ of the amorphous region in PP. this shows that the compatibility of IIR and PP is poor at this time, and phase separation is obvious. According to the phase evolution during DV, the uncross-linked IIR phase wraps the PP phase when the DV time is 10 s. Rubbers and plastics have larger phase sizes, and IIR/PP-TPVs exhibit two distinct T_g_. When phase inversion begins (DV time reaches 30 s), IIR/PP-TPV transitions to the continuous phase and exhibits two T_g_. When the DV reaction is completed, IIR cross-linking particles are dispersed in the PP matrix, and IIR/PP-TPV exhibits only one tan δ. The tan δ peak of the IIR moves towards a higher temperature and the tan δ of the PP phase became weak, indicating that the compatibility of rubber and plastic improved. This occurs because, at the end of DV, IIR/PP-TPV forms a sea–island morphology with the IIR cross-linking particles as the dispersed phase and with PP as the continuous phase. The IIR cross-linking particles uniformly dispersed around the PP phase destroyed the amorphous region of PP, resulting in the disappearance of the T_g_ of PP, which increases the compatibility of the rubber–plastic phase [26].

The damping property of rubber material is related to its dynamic mechanical relaxation and depends on its hysteresis phenomenon. The damping temperature range (T_p_) is represented by the absolute value of temperature between two points, tan δ ≥ 0.3 in the DMA thermogram [27]. The T_p_ of the IIR/PP-TPV at different DV times is listed in Table 1. Table 1 shows that the T_p_ of IIR/PP-TPV increases with an increase in DV time, indicating that the T_p_ of the IIR/PP-TPV gradually widens. The cross-linking degree of the TPV gradually increases with the progress of DV, which restricts the movement of macromolecular segments in the IIR and causes T_g_ to move towards higher temperatures. The IIR phase was broken into IIR cross-linking particles (about 2 μm) and dispersed in the PP phase so that IIR/PP-TPV evolved into sea–island morphology. The spherulite size of the PP decreases and the distribution becomes more uniform. In this manner, the sea–island morphology of the IIR/PP-TPV can absorb more thermal energy, showing wider damping temperature ranges and better damping performance.

### 3.5. Recyclability

**Figure 7:**
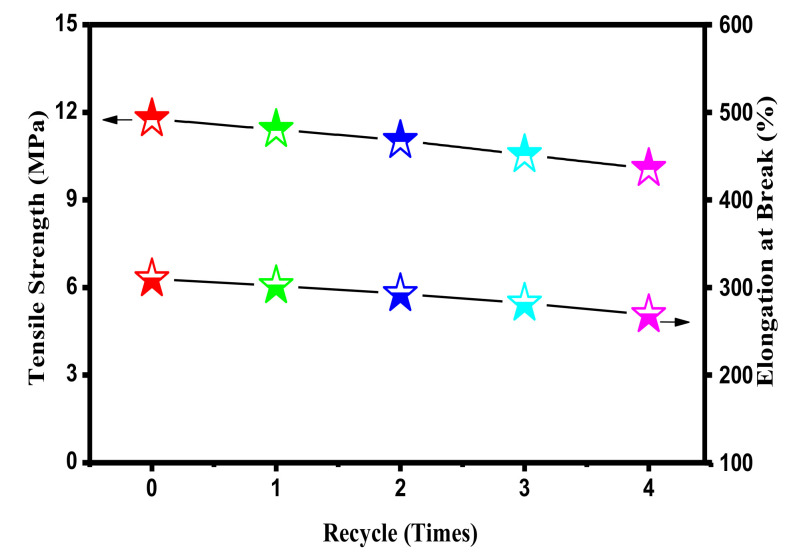
Mechanical properties of repeatability of IIR/PP-TPV with different recycling times.

The recyclability of the IIR/PP-TPV used in shock-absorption and sound-insulation devices can protect the environment. Meanwhile, the recyclability of the IIR/PP-TPV can save resources and energy. The changes in physical properties and microscopic phase of sample (DV time reaches 120 s) after different recycle times are used to measure the recyclability of IIR/PP-TPV. In order to simulate recycling conditions, the sample was collected after tensile testing (broken samples) and placed at room temperature for 24 h; then, they were dried in an oven at 50 °C for 12 h. The broken samples were melt-reconstituted in a torque rheometer (180 °C × 60 rpm·min^−1^ × 3 min); then, 2 mm thick sheets were prepared by molding for tensile property testing. We conducted four recycling experiments. The tensile strength and elongation at break are shown in Figure 7. These experiments showed that the retention rate of tensile strength and the elongation at break of the IIR/PP-TPV reached 88% and 86%, respectively, indicating that IIR/PP-TPV still has good physical properties after four recycling cycles.

**Figure 8:**
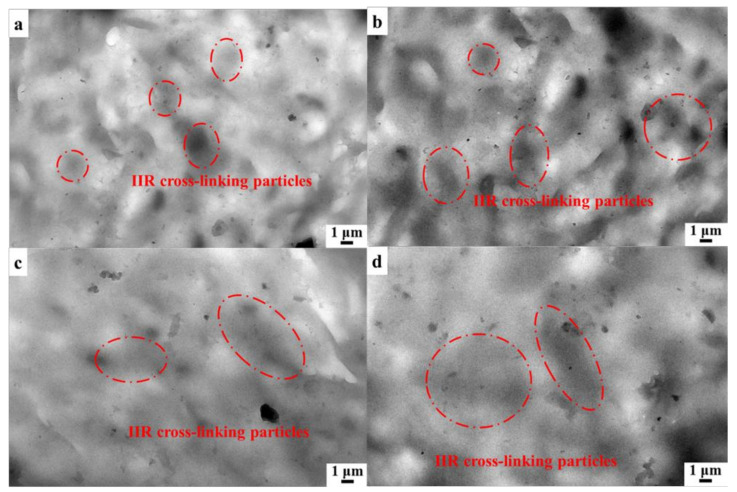
TEM micrographs of IIR/PP-TPV samples with different recycling times: (**a**) 0 times, (**b**) 1 time, (**c**) 3 times, and (**d**) 4 times.

In order to further clarify the mechanism of IIR/PP-TPV recycling, we observed the microscopic phase of the samples. Figure 8 shows the TEM micrographs of the IIR/PP-TPV samples with different recycling times. Figure 8 shows that the IIR/PP-TPV presents obvious sea–island morphology. The IIR cross-linking particles were still surrounded by the PP phase after four cycles, indicating that the continuous PP phase provides repeated processability for IIR/PP-TPV. The size of the IIR cross-linking particles in the IIR/PP-TPV (Figure 8a) is about 2 μm, which is consistent with the SEM micrographs shown above. The dispersed rubber phase shows the smallest size and more uniform size distribution, and the resulting IIR/PP-TPV demonstrates the best mechanical properties. A similar result can be found in the literature. For example, Ning et al. investigated the microstructure and properties of BIIR/PA12-TPV. They found that the reduction in the BIIR phase was beneficial to improve the tensile strength of BIIR/PA12-TPV [28]. Zhao et al. reported that the particle size of EPDM rubber aggregates in EPDM/PP-TPV obtained by conventional injection molding was about 1.5 μm [29]. When the sample was recycled three times and four times, the particle size was about 5 μm. The size of the IIR cross-linking particles in IIR/PP-TPV increased after four cycles of recycling, mainly because the tensile strain of the IIR/PP-TPV was caused by the slip and orientation deformation of the rubber phase [30]. The rubber cross-linking network cannot be recovered at high temperature, but the deformation of the PP phase can be recovered above the melting temperature [31]. Importantly, the prepared IIR/PP TPV exhibits excellent recyclability, high elasticity, and good damping property.

## 4. Conclusions

We successfully prepared IIR/PP-TPV by DV and studied the morphological evolution during DV and the properties of the IIR/PP-TPV. The as-prepared IIR/PP-TPV exhibits excellent recyclability, high elasticity, and good damping property. The prepared IIR/PP-TPV has sea–island morphology, and the size of the IIR cross-linked particles reaches 2 μm, demonstrating good recyclability, high elasticity, and good damping performance. The mechanism for the formation of the phase morphology and morphological evolution during DV and the microstructure property relationship of IIR/PP-TPV were deeply studied. The results indicate that the change of cross-linking degree during DV has an important effect on the morphological evolution, damping properties, and physical properties of IIR/PP-TPV. The T_p_ of the IIR/PP-TPV broadened with the disappearance of T_g_ in the PP phase, ascribed to the improvement of compatibility between the IIR and PP with increasing DV time. As DV progresses, the size of the dispersed IIR particles and PP crystalline phase decreases, leading to the formation of a sea–island morphology. The size of the IIR cross-linked particles in the IIR/PP-TPV becomes larger after melt-recombination, and the continuous PP phase provides excellent recyclability. DV is an important preparation method of TPV that affects the relationship between the microstructure and properties. This study provides guidance for the preparation of high performance IIR/PP-TPV by controlling the microstructure.

## Figures and Tables

**Table 1:** T_p_ and T_g_ of IIR/PP-TPV with different DV times.

Sample	T_p_ (°C)	T_g_ of IIR (°C)	T_g_ of PP (°C)
10 s	20.2	−51.3	2.3
30 s	25.9	−47.9	2.5
90 s	34.6	−43.7	-
120 s	43.6	−40.6	-

## Data Availability

Not applicable.
